# What is the impact of the COVID-19 pandemic on residency training: a systematic review and analysis

**DOI:** 10.1186/s12909-021-03041-8

**Published:** 2021-12-15

**Authors:** Shou-Yen Chen, Hsiang-Yun Lo, Shang-Kai Hung

**Affiliations:** 1 Department of Emergency Medicine, Linkou Chang Gung Memorial Hospital, 333 Taoyuan City, Taiwan; 2 Graduate Institute of Clinical Medical Sciences; Division of Medical Education, College of Medicine, Chang Gung University, 333 Taoyuan City, Taiwan

**Keywords:** COVID19, Residency training, Residents, Medical education

## Abstract

**Background:**

The coronavirus disease 2019 (COVID-19) pandemic has greatly affected medical education in addition to clinical systems. Residency training has probably been the most affected aspect of medical education during the pandemic, and research on this topic is crucial for educators and clinical teachers. The aim of this study was to understand the effect of the COVID-19 pandemic comprehensively through a systematic review and analysis of related published articles.

**Methods:**

A systematic review was conducted based on a predesigned protocol. We searched MEDLINE and EMBASE databases until November 30, 2020, for eligible articles. Two independent reviewers extracted data by using a customized form to record crucial information, and any conflicts between the two reviewers were resolved through discussion with another independent reviewer. The aggregated data were summarized and analyzed.

**Results:**

In total, 53 original articles that investigated the effect of the COVID-19 pandemic on residency training were included. Studies from various regions were included in the research, with the largest percentage from the United States (*n* = 25, 47.2%). Most of these original articles were questionnaire-based studies (*n* = 44, 83%), and the research target groups included residents (79.55%), program directors (13.64%), or both (6.82%). The majority of the articles (*n* = 37, 84.0%) were published in countries severely affected by the pandemic. Surgery (*n* = 36, 67.92%) was the most commonly studied field.

**Conclusions:**

The COVID-19 pandemic has greatly affected residency training globally, particularly surgical and interventional medical fields. Decreased clinical experience, reduced case volume, and disrupted education activities are major concerns. Further studies should be conducted with a focus on the learning outcomes of residency training during the pandemic and the effectiveness of assisted teaching methods.

**Supplementary Information:**

The online version contains supplementary material available at 10.1186/s12909-021-03041-8.

## Background

The coronavirus disease 2019 (COVID-19) pandemic has greatly affected every facet of the health care system since COVID-19 was first identified in Wuhan, China, in December 2019. The pandemic overwhelmed health care systems rapidly in many countries, and numerous studies have investigated the clinical effects of the pandemic [1–5]. The COVID-19 pandemic has not only damaged clinical systems but also affected medical education severely. However, research on the effect of the COVID-19 pandemic on education is as-yet limited.

When considering medical education, residency training has probably been the most affected during the COVID-19 pandemic because the core of residency training is clinical experience and clinical skill proficiency, which have been reduced because of multiple factors in the pandemic. Dedeilia et al. published a systematic review on educational challenges in the COVID-19 era and revealed that both medical and surgical education have been severely affected [6]. However, the article was published in the early stage of the pandemic when literature on the topic was limited, and the focus of the research was not only residency training but also medical students’ education. Additionally, most of the included articles were nonoriginal manuscripts and may have been insufficient to analyze the effect of the COVID-19 pandemic on residency training.

Understanding the influence of the pandemic on residency training is crucial to being able to adopt methods to maintain consistency in training quality. The aim of this study was to identify the real effect of the COVID-19 pandemic on residency training through a systematic review of relevant published articles and further analysis of the results. Our study may help residency training program directors worldwide to comprehensively understand the influence of the pandemic and adopt assisted teaching methods to provide effective training.

## Methods

### Systematic review protocol

A systematic review was conducted based on a predesigned protocol in accordance with the Preferred Reporting Items for Systematic Reviews and Meta-Analyses (PRISMA) statement [7].

### Search strategy

We searched the MEDLINE and EMBASE databases for eligible articles until November 30, 2020 (for approximately 1 year from the commencement of the COVID-19 pandemic). The search strategy was based on the following algorithm: (“COVID- 19” [All Fields] OR “COVID-2019” [All Fields] OR “severe acute respiratory syndrome coronavirus 2” [Supplementary Concept] OR “severe acute respiratory syndrome coronavirus 2” [All Fields] OR “2019-nCoV” [All Fields] OR “SARS-CoV-2” [All Fields] OR “2019nCoV” [All Fields] OR (“Wuhan” [All Fields] AND (“coronavirus” [MeSH Terms] OR “coronavirus” [All Fields]))) AND (“education, medical” [MeSH Terms] OR (“education” [All Fields] AND “medical” [All Fields]) OR “medical education” [All Fields] OR “residency training” [All Fields] OR “trainee” [All Fields]).

### Inclusion and exclusion criteria

We aimed to include all original articles discussing the effect of COVID-19 on residency training in different specialties. Furthermore, we only included studies that were published in English. We excluded nonoriginal articles, such as reviews, editorials, perspectives, short or special communications, and letters to editors. Studies regarding medical students or paramedical specialties, such as dentistry or nursing, were excluded. Moreover, studies focused on methodologies or technological innovations rather than residency training were excluded.

### Study selection and data extraction

**Fig. 1:**
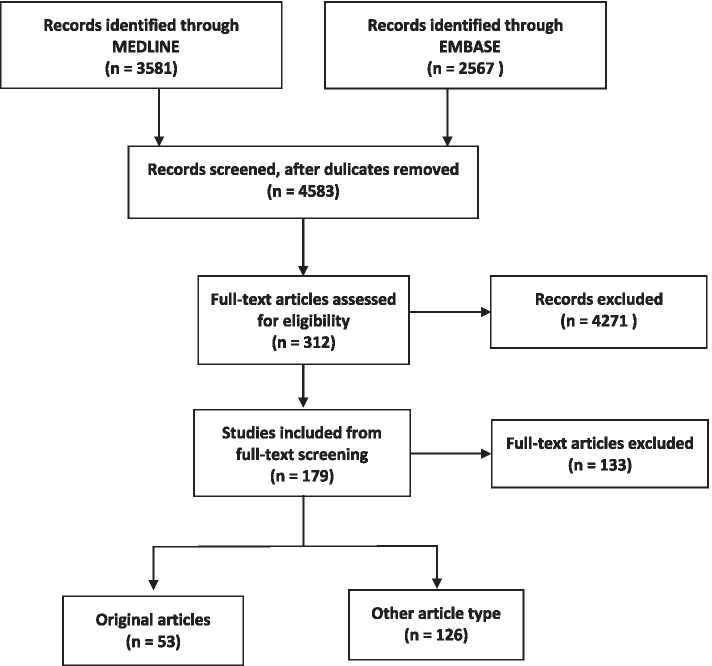
PRISMA flow diagram of the study selection process

Figure 1 depicts the study selection and review processes. After selecting articles from two databases, we removed duplicate articles manually. Two independent reviewers (SKH and HYL) scanned the title and abstract of all articles to determine relevancy in light of the inclusion/exclusion criteria. Articles without abstracts were included for full-text assessment and evaluated at that stage. Two reviewers shared their results after scanning the title and abstract of all articles, and only articles that were excluded by both reviewers were eliminated from further full-text assessment. During the full-text assessment stage, we used a customized Excel sheet to record essential information of the article. Article type, first author of the article, training specialties, publication date, target group, and country of the enrolled population were extracted. We classified training specialties into four categories. Surgical field including otorhinolaryngology, orthopedics, general surgery, neurosurgery, obstetrics and gynecology, oral and maxillofacial surgery, cardiothoracic surgery, urology, ophthalmology and oculoplastic surgery. Medical filed including internal medicine, diagnostic radiology, pediatrics, emergency medicine and pathology. Interventional field including interventional cardiology, interventional radiology and endoscopy.

Then, two reviewers evaluated the extracted full-text articles separately according to the criteria. Any conflicts between the two reviewers regarding the extracted articles were resolved through discussion with another reviewer (SYC). The included articles were further quantitatively analyzed or described narratively.

### Statistical analysis

Descriptive statistics of aggregated data are presented using count and proportions. Descriptive analysis was performed according to publication date, country, and specialty separately. Further subanalysis was performed based on study content, geographic location, pandemic severity, and specialty category. Pandemic severity was defined according to the World Health Organization dashboard [8]. Statistical analysis was performed using Microsoft Excel (2016, Microsoft Corporation, Seattle, Washington).

## Results

The search yielded 3581 and 2567 articles from MEDLINE and EMBASE, respectively. After removing duplicates, 4583 articles were left; their titles and abstracts were scanned, and 312 relevant articles were identified. The full texts of these 312 articles were reviewed with 179 articles included. Among the included articles, 53 original articles were undergone further data extraction and quantitative analyses. The key information of the 53 original articles is summarized in Table 1 and Additional file 1, with the articles presented in alphabetical order by name of first author.

**Table 1:** Key information of the included articles

No.	First author (Date)	Study specialty	Type of study	Target of the questionnaire	Cases-count of country where study was carried out (cases)	Main findings
1	Abdul Hafiz Oladapo Adesunkanmi (Oct. 2020) [9]	Surgery	Questionnaire	Residents	50,001–500,000	The COVID-19 pandemic has significantly affected the clinical, research and teaching components of surgical training in Nigeria.
2	Adel Salah Alahmadi (Nov. 2020) [10]	Ophthalmology	Questionnaire	Residents	50,001–500,000	COVID-19 pandemic has disrupted residents’ clinical and surgical training and may have a negative impact on residents’ mental health.
3	Ahmad K Alhaj (May 2020) [11]	Neurosurgery	Questionnaire	Residents	Multiple countries	Neurosurgery residents have a relatively good knowledge about COVID-19 but most participants did not receive sufficient training about PPE.
4	Ahmed M Gabr (Jun. 2020) [12]	Diagnostic and interventional radiology	Non- questionnaire	–	> 1,000,000	COVID-19 pandemic related decreased case volume and cancellation of conferences had significant impact on diagnostic and interventional radiologist residency training.
5	Ameera Balhareth (Jul. 2020) [13]	Multiple specialties	Questionnaire	Residents	50,001–500,000	The slowdown of residents’ and fellows’ learning curve is inevitable, so the adoption of smart learning is critical. Anxiety and depression were noticed upon enrolled residents during the pandemic.
6	Anne L Cravero (Sep. 2020) [14]	Multiple specialties	Questionnaire	Residents	Multiple countries	Exposure to patients with COVID-19 is significantly associated with higher burnout rates in residents.
7	Basil M Kahwash (Sep. 2020) [15]	Allergy and Immunology	Questionnaire	Residents	USA and Canada	Majority of fellows described substantial changes in daily activity such as reassignment to other services. The positive impact of telemedicine on resident education were also reported by enrolled fellows.
8	Cesare Zoia (Jun. 2020) [16]	Neurosurgery	Questionnaire	Residents	> 1,000,000	Most enrolled neurosurgical residents reported significant reduction in clinical surgical activity but increased in educational and scientific activities.
9	Christoph Roemmele (May 2020) [17]	Endoscopy	Non-questionnaire	–	> 1,000,000	COVID-19 outbreak has a negative impact on endoscopy training of gastroenterology fellows in a high-volume center in Germany.
10	Davide Pertile (Jun. 2020) [18]	Surgery	Questionnaire	Residents	> 1,000,000	COVID-19 pandemic has severely impacted the educational program of surgical residents in Italy.
11	Deepak Mishra (May 2020) [19]	Ophthalmology	Questionnaire	Residents	> 1,000,000	Most participants felt that the COVID-19 lockdown adversely affected their learning, especially surgical training while most found online classes and webinars useful. The residents’ perceived stress levels were higher than normal during the lockdown.
12	Devang Odedra (Jun. 2020) [20]	Diagnostic and interventional radiology	Questionnaire	Residents	500,001–1,000,000	The COVID-19 pandemic has had a significant impact on various domains of the radiology residency programs, which has been mitigated by several new technology-based strategies.
13	Dong-Gune Chang (Jul. 2020) [21]	Orthopedics	Questionnaire	Residents	5001–50,000	COVID-19 pandemic had a significant impact on orthopedic residency training on decreased clinical experience and educational activity in South Korea.
14	E Christopher Ellison (Aug. 2020) [22]	Surgery	Questionnaire	Residents	> 1,000,000	The pandemic has forced innovation in clinical experiences and didactic learning to deal with the adverse impact on residency training.
15	Edward J Caruana (Oct. 2020) [23]	Cardiothoracic surgery	Questionnaire	Residents	> 1,000,000	Most of the respond cardiothoracic surgical resident were concerned about their physical and mental health. Redeployment, inadequate PPE, decreased case volume and multi-disciplinary team meetings are major concerns.
16	Erin M White (Jul. 2020) [24]	General surgery	Questionnaire	Program directors	> 1,000,000	Program directors reported dramatically reduced in face-to-face learning opportunities for surgical residents during the COVID-19 pandemic and all programs initiated some strategies to protect resident health.
17	Francesco Bandi (Jul. 2020) [25]	Otorhinolaryngology	Questionnaire	Residents	> 1,000,000	Surgical training has been reported by enrolled residents as the activity perceived to be the most contracted during the pandemic.
18	Garrett N Coyan (Aug. 2020) [26]	Thoracic surgery	Questionnaire	Program directors	> 1,000,000	Thoracic surgery program directors reported several changes in the delivery of training program including decreased operative experience, but most participants are confident that graduating thoracic surgery residents will finish on time.
19	Gaurav Kumar Upadhyaya (Jul. 2020) [27]	Orthopedics	Questionnaire	Residents	> 1,000,000	COVID-19 pandemic has severely disrupted the education and training of the orthopedic residents in India.
20	Geoffrey H Rosen (May 2020) [28]	Urology	Questionnaire	Program directors	> 1,000,000	Residents have more time away from urology related clinical duties including redeployment and wellness is a priority for program directors.
21	Giovanna Bitonti (Jul. 2020) [29]	Gynecology	Questionnaire	Residents	> 1,000,000	COVID-19 pandemic had a considerable negative impact on obstetrics and gynecology residency training program and new strategies are necessary to minimize training deficiencies.
22	Hassan Aziz (Jul. 2020) [30]	General surgery	Questionnaire	Residents	> 1,000,000	COVID-19 has significant impact on surgical training and education. Except to negative impact, a positive consequence of the pandemic is increased educational didactics and should be continued after the pandemic.
23	Hsiang-Yun Lo (Sep. 2020) [31]	Emergency medicine	Non- questionnaire	–	1–5000	The COVID-19 pandemic engendered a reduced emergency department volume and decreased emergency medicine residents’ clinical exposure. All portion of emergency medicine residency training were affected by the pandemic, with pediatric emergency medicine being the most affected.
24	Jared Johnson (Jun. 2020) [32]	Pediatric Otorhinolaryngology	Questionnaire	Program directors	> 1,000,000	Pediatric otolaryngology fellowship directors reported the COVID-19 pandemic has had a significant impact on training program, with the majority feeling that both their fellows surgical and clinical experience have been significantly impacted.
25	Jessica B Robbins (Jun. 2020) [33]	Diagnostic and interventional radiology	Questionnaire	Program directors	> 1,000,000	Enrolled program directors reported COVID-19 pandemic has markedly impacted the perceived well-being and educational missions of radiology residency programs across the U.S.
26	Jian Zheng (Jun. 2020) [34]	General surgery	Questionnaire	Residents	> 1,000,000	Enrolled chief residents were not as concerned with the case requirements for board examination but were more concerned in the potential delay in the date of the examinations and inadequate preparedness for these examinations.
27	Johnathan A Khusid (May 2020) [35]	Urology	Questionnaire	Residents	> 1,000,000	Advocating for adequate PPE, providing support at the residency program and institutional levels, telehealth education programs and fostering a sense of shared responsibility for COVID-19 patients may optimize urology residents’ well-being and education.
28	Joshua D Burks (Sep. 2020) [36]	Neurosurgery	Non-questionnaire	–	> 1,000,000	Impact of the COVID-19 pandemic on neurosurgery residency training is different from levels of residents and areas of the surgery.
29	Julia R Coleman (Sep. 2020) [37]	Surgery	Questionnaire	Residents	> 1,000,000	COVID-19 pandemic has a significant impact on the lives of surgical residents. Increased PPE access and wellness resources targeting in high-risk groups are actionable items to response for major health crisis during the pandemic.
30	Katarzyna M Pawlak (Jun. 2020) [38]	Gastrointestinal Endoscopy	Questionnaire	Residents	Multiple countries	COVID-19 pandemic has led to restrictions in endoscopic volumes and endoscopy training, with high rates of anxiety and burnout among endoscopy residents worldwide.
31	Katherine E Fero (May 2020) [39]	Urology	Questionnaire	Residents and program directors	> 1,000,000	Enrolled participants reported decreased case volume, increased use of telehealth and educational activities via virtual platforms. Program directors and residents perceive an overall negative impact on surgical training and increased anxiety about competency.
32	Khurram Shahzad Khan (Aug. 2020) [40]	Surgery	Questionnaire	Residents	> 1,000,000	COVID-19 pandemic has an unprecedented negative impact on all aspects of core surgical training.
33	Kofi Clarke (Oct. 2020) [41]	Gastrointestinology	Questionnaire	Residents	> 1,000,000	COVID-19 pandemic has impacted GI fellowship training in the U.S. in multiple aspects, including endoscopy, inpatient and outpatient activities, and educational conferences.
34	Madhusudan Ganigara (Oct. 2020) [42]	Pediatric cardiology	Questionnaire	Residents	> 1,000,000	Restrictions imposed by the COVID-19 pandemic have greatly increased utilization of online learning platforms. This survey reveals that an online lecture curriculum offers advantages that may mitigate some negative consequences of the pandemic.
35	Mariantonia Ferrara (Jun. 2020) [43]	Ophthalmology	Questionnaire	Residents	Multiple countries	The vast majority of residents reported decreased of clinical and surgical activities. Application of new technology-based tools is needed.
36	Medalit E Huamanchumo-Suyon (Oct. 2020) [44]	General surgery	Questionnaire	Residents	> 1,000,000	The impact of COVID-19 has been reflected in a decreased number of elective surgical procedures, daily activities in the surgery department and the suspension of rotations. The majority of residents reported that the impact of COVID-19 in their training was severe.
37	Melissa K Meghpara (Nov. 2020) [45]	Surgery	Non- questionnaire	–	> 1,000,000	Repurposing the surgical department for the concerns of the pandemic has involved all surgical staff. The strengths of the repurposing allowed the team to meet the demands posed by the pandemic.
38	Muhammad Osama (Jun. 2020) [46]	Surgery	Questionnaire	Residents	500,001–1,000,000	Reduction in working hours, hands-on and clinical case volume were found during the pandemic in surgical residency training. Psychological burdens of fear of acquiring the infection should be addressed.
39	Nahuel Paesano (Jul. 2020) [47]	Urology	Questionnaire	Residents	Multiple countries	COVID-19 pandemic is negatively impacting the residency programs. It is necessary to continue with technological innovation and allocate time and resources to generate accessible tools for future training.
40	Natalie A Homer (Oct. 2020) [48]	Oculoplasty	Questionnaire	Residents	> 1,000,000	During the COVID-19 restrictions most participated in emergent clinical activities and novel telemedicine curriculum. Most residents expressed concern regarding a negative impact on overall subspecialty education and surgical confidence.
41	Neo Poyiadji (Sep. 2020) [49]	Diagnostic radiology	Non- questionnaire	–	> 1,000,000	COVID-19 pandemic caused a marked decrease in radiology resident imaging interpretation volume and has had a tremendous impact on resident education.
42	Paloma Del C Monroig-Bosque (Jul. 2020) [50]	Pathology	Non- questionnaire	–	> 1,000,000	Redeploying pathology residents during the COVID-19 pandemic resulted in optimization of patient care while ensuring residents’ safety and helped to maintain continuous high-quality education through active involvement in unique learning opportunities.
43	Panayiotis D Megaloikonomos (Jul. 2020) [51]	Orthopedics	Questionnaire	Residents	Multiple countries	Most respond residents in Europe felt the decrease in clinical, surgical and educational activities would have a great effect on their training in orthopedic and trauma field.
44	Panayiotis E Pelargos (Aug. 2020) [52]	Neurosurgery	Questionnaire	Residents	Multiple countries	All enrolled residents have experienced reduced work hours and a reduction in operative case volumes. Adaptions including increased didactic time and using electronic platforms.
45	Raphael E Huntley (Aug. 2020) [53]	Oral and Maxillofacial Surgery	Questionnaire	Residents	> 1,000,000	COVID-19 pandemic has great impact on oral and maxillofacial surgery residency training. Residents’ concerns on availability of PPE, operative experience and completion of graduation requirements.
46	Robert J Rothrock (Jul. 2020) [54]	Neurosurgery	Non- questionnaire	–	> 1,000,000	During the height of the COVID-10 pandemic, 59.4% reduction in daily neurosurgical census over the study period and 83.8% reduction in case volume compared with similar period in 2019.
47	S Veerasuri (Jul. 2020) [55]	Diagnostic and interventional radiology	Questionnaire	Residents	> 1,000,000	During the pandemic, there have been significant changes to departmental workflows, radiology workload, and the nature of daily work thus had a significant impact on training and residents’ well-being.
48	Samit Shah (Aug. 2020) [56]	Interventional cardiology	Questionnaire	Residents and program directors	> 1,000,000	The pandemic affected interventional cardiology fellowship training in the U.S. with many fellows at risk of not satisfying program procedural requirements.
49	Tanush Gupta (May 2020) [57]	Interventional cardiology	Questionnaire	Residents and program directors	> 1,000,000	The pandemic caused a marked volume reduction in cardiac catheterization laboratory and impacted interventional cardiology fellowship training.
50	Theresa Guo (Jun. 2020) [58]	Otolaryngology	Questionnaire	Residents	Multiple countries	The majority of respond residents felt that their education and training had been negatively impacted by the pandemic in particular in surgical training.
51	Tonya W An (Aug. 2020) [59]	Orthopedics	Questionnaire	Residents	> 1,000,000	Programs currently use a variety of strategies to provide essential orthopedic care. Continued prioritization of resident safety and necessary training accommodations is needed.
52	Virginia K Singla (Nov. 2020) [60]	Cardiovascular Electrophysiology	Questionnaire	Program directors	> 1,000,000	The COVID-19 pandemic has resulted in a decrease in procedural volume for clinical cardiac electrophysiology procedures, but the majority of the participants do not anticipate major barriers to timely graduation.
53	Zaid S Aljuboori (Oct. 2020) [61]	Neurosurgery	Non- questionnaire	–	> 1,000,000	A significant reduction in operative volume in neurosurgery residency training programs. Increased research-related activities and research productivity were also reported.

### Quantitative analyses of original articles

**Fig. 2:**
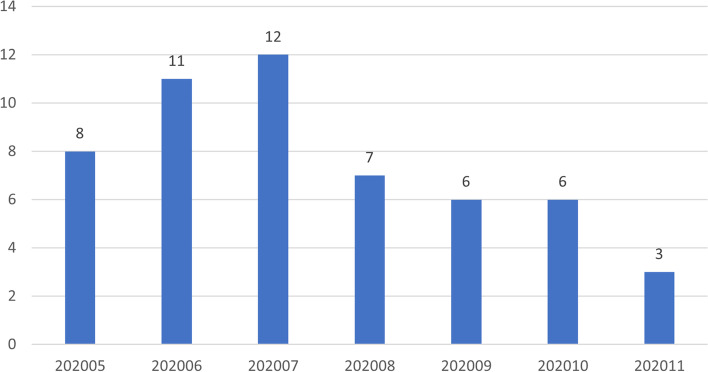
Time distribution of the published articles

**Fig. 3:**
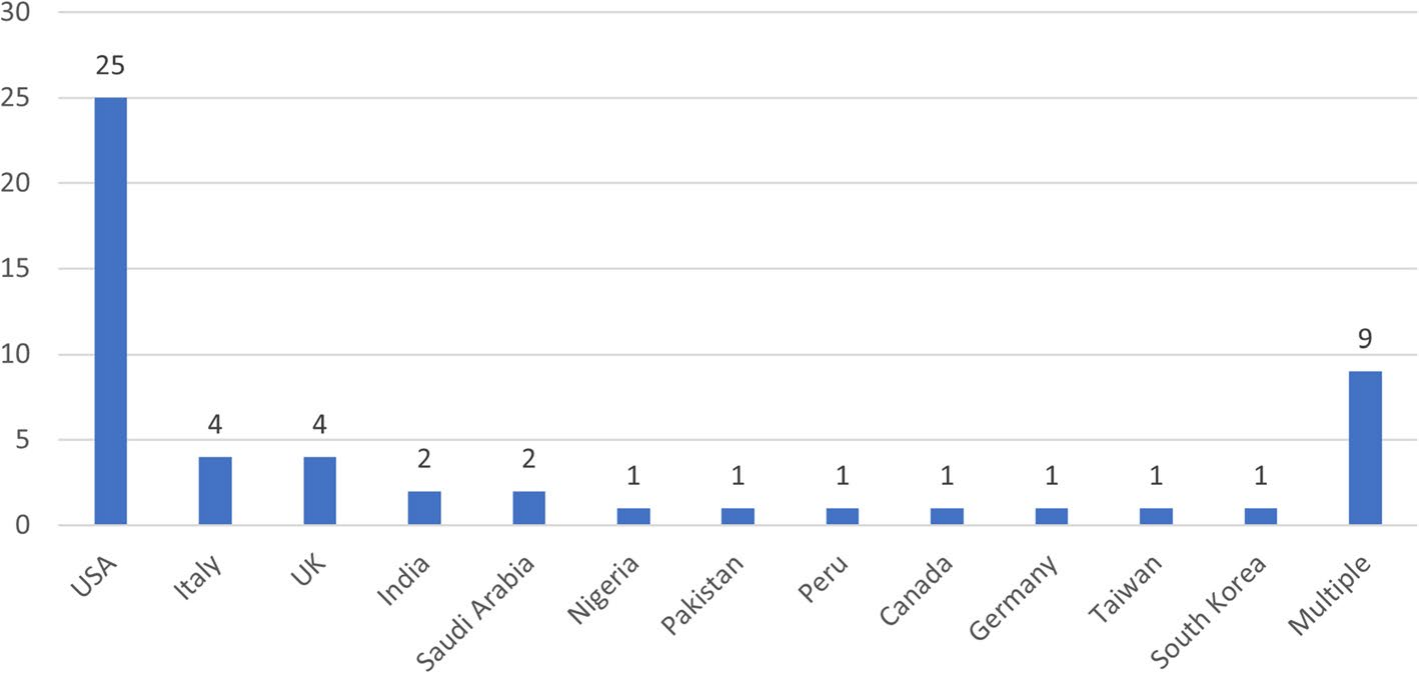
Geographic distribution of the published articles

Of the 53 original articles, 8, 11, 12, 7, 6, and 3 articles were published in May, June, July, August, September and October, and November 2020, respectively (Fig. 2). The geographic distribution of the research target group of the articles is presented in Fig. 3. By country, the most studies were performed in the United States (25 articles), followed by Italy and the United Kingdom (4 articles). Most studies focused on residency training in a single country, and only nine articles involved residents of multiple countries as the target group.

### Subgroup analyses of original articles

Most of these original articles were questionnaire-based studies (*n* = 44, 83%). Among questionnaire-based studies, 35 (79.6%) involved residents directly, 6 (13.6%) involved program directors, and 3 (6.8%) involved both groups. The distribution of published articles according to the degree of the COVID-19 pandemic was demonstrated as following: 37 (84.0%), 2 (4.6%), and 3 (6.8%) articles were published in countries with > 1,000,000, 500,001–1,000,000, and 50,001–100,000 cases, respectively. Table 2 presents studies according to specialty. Most articles focused on surgery (*n* = 36, 67.92%) and interventional skill training in different specialties (*n* = 9, 16.98%).

**Table 2:** Distribution of included articles by specialty

Study field	Article number (n)	%
Surgical	36	67.92
Medical	6	11.32
Interventional	9	16.98
Multiple	2	3.77

### Possible effects of the COVID-19 pandemic on residency training

We summarize the effect of the COVID-19 pandemic of residency training mentioned in these articles in Table 3. The most common effect of the pandemic on residency training was decreased clinical experience and failure to meet the training requirements of the specialty. Change in the working hours of residents varied based on hospital and specialty. Some articles reported decreased working hours because of reduced patient volume and elective operations. However, some residents may have experienced increased burdens because of extra work due to the pandemic. Educational activities such as lectures or case discussions may have increased or decreased depending on the situation. Furthermore, some articles mentioned redeployment to other tasks to manage the COVID-19 pandemic and some COVID-19-related problems, such as inadequate personal protective equipment (PPE) and quarantine policies. Moreover, residents had worse mental health and increased anxiety regarding their board exams and career.

**Table 3:** Summary of the effect of the COVID-19 pandemic on residency training

Theme	Number of included original articles mentioned (N)
Decreased clinical experience and reduced case volume	51 (96.23%)
Working hours and burnout: may increase or decrease	33 (62.26%)
Educational activity: may increase or decrease (lectures and case discussions)	28 (52.83%)
COVID-19-related problems: inadequate personal protective equipment, quarantine policies	18 (33.96%)
Redeployment to manage the COVID-19 pandemic	14 (26.41%)
Failure to meet training requirements	12 (22.64%)
Anxiety regarding board exams and career	7 (13.21%)
Decreased quality of life and worse mental health	6 (11.32%)

## Discussion

Our study is the first to systematically review and analyze published academic articles focusing on the effect of the COVID-19 pandemic on residency training after 1 year of the pandemic. We provided statistical information on the publication timeline, geographic distribution, publication types, and study specialties. In addition, we summarized the effect of the COVID-19 pandemic on residency training narratively.

The World Health Organization declared COVID-19 a pandemic on March 11, 2020. With respect to the global accumulation of cases, the number of confirmed patients with COVID-19 exceeded 1 million on April 2, and the number of deaths due to COVID-19 exceeded 100,000 on April 10 [62]. The first original article on residency training during the pandemic was published on May 16, and publication on this topic peaked in July with 12 articles [57]. This timeline reflects rapid intensification of the global pandemic in this period, which affected residency training in many specialties. Additionally, several developed countries, such as the United States, Italy, and the United Kingdom, had a rapidly increasing number of COVID-19 cases at this time, which may have been the reason for the increasing number of studies on this topic in these countries, given their focus on research and medical education. Although number of affected cases in countries apart from Americas and Europe is high, the publications carried out from these regions are disproportionately low. It may relate to research energy of individual country, publication bias or selection bias from our inclusion language criteria. Owing to lack of adequate publications, we cannot make a comprehensive between-country comparison of the impact on residency training and further investigation is warranted in the future.

Regarding geographic distribution, the studies were globally distributed, having been conducted in both developed or developing countries, and several multinational studies were also noted. Although the United States was responsible for almost half the articles reviewed, the results suggested that residency training programs were being affected globally by the COVID-19 pandemic. The effect of the pandemic on residency training should be identified by residency training program directors, and rolling adjustments and training program revisions are necessary to provide adequate training and maintain consistent quality during the pandemic.

The majority of the studies were published in countries severely affected by the pandemic, and therefore, medical education and residency training were substantially affected in these countries. Fewer elective operations because of policies established during the pandemic were noted in some specialties, such as urology, orthopedics, plastic surgery, and diagnostic angiography [12, 63–65]. The policies of lockdown and quarantine reduced patient volume for some diseases, such as trauma and infectious diseases, which induced less clinical exposure [66, 67]. Redeployment and reassignment of residents occurred for pandemic-related work [41]. All these factors contributed to possible inadequate training and failure to meet the requirements of training programs.

Surgical specialties were the most and first affected. Elective surgeries were cancelled or postponed during the pandemic, providing less practice and experience for the residents [63–65, 67]. Additionally, residents in surgical specialties were redeployed to manage patients with COVID-19 or perform other work due to medical resource reallocation, disrupting their original surgical training courses. Another disruption was the “stay at home” policy, which resulted in fewer trauma cases and related surgeries [68]. All these factors affected surgical resident training. A similar situation occurred in interventional medical fields, such as radiology, cardiology, and gastroenterology [12, 69, 70]. Hands-on practice and on-the-job skill development in residency training were affected during the pandemic.

Although the effect of COVID-19 on the internal medicine field was rarely mentioned in the articles, the influence still existed. Residents focused on treating patients with COVID-19, which decreased their clinical experience with other diseases [15]. The fear of becoming infected with COVID-19 in hospitals reduced patient volume, except for patients with possible or confirmed COVID-19 [71]. Moreover, the policy of wearing a mask decreased the incidence of infectious diseases such as influenza [72]. These conditions occurred in many countries, even in those less severely affected by the pandemic. Lo et al. published an observational study on the effect of the COVID-19 pandemic on emergency medicine residency training in a teaching hospital in Taiwan [31]. As of this writing, in Taiwan, the number of patients with confirmed COVID-19 has remained under 1000, no large-scale lockdown has been implemented, and the health care system is robust. However, significant decreases were nevertheless noted in case volume and residents’ clinical exposure in Taiwan. Yet, the real effect of decreased clinical exposure is unknown because no study has assessed the learning outcomes of residents undergoing training during the pandemic. Other than negative impact, studies also revealed some positive impact worth mentioning and included implementation of new technology educational tools, higher quality of live-streamed didactic lectures, broader applications of telemedicine, increased time on research activities and self-study. A study collected narrative impressions revealed some surgical residents considered the pandemic is a valuable chance to learn deeper on communicable diseases and to think of the entire body again rather than separate body organs.

The mental health and quality of life of residents have potentially been affected. The psychological pressure has been heavy when managing patients with COVID-19 because of infection risk and inadequate PPE in some hospitals. Quarantining due to caring for patients with confirmed COVID-19 and the fear of infecting family led residents to live alone with little family support [14]. Excessive working hours and burnout have been common, although these factors have varied by individual. These factors can induce a stressed and unhealthy mental state and impair learning [30].

According to our results, most of the original studies used questionnaire surveys. In the early stage of the pandemic, the advantage of this research type was that it was quick, saved time, and was easily accessible [73]. The results of these questionnaire surveys with either residents or program directors as respondents exhibited high consistency and reflected the effect of the COVID-19 pandemic on residents. However, these questionnaire studies had some limitations, such as subjective questions, voluntary response bias, and heterogeneity of respondents and institutional bias, which may limit their generalizability [74]. Only nine studies had conducted objective investigations and had “real numbers” comparing the prepandemic and pandemic periods [17, 31, 36, 39, 45, 49, 50, 54, 61]. These studies indicated the severity of the effect and identified domains that were affected. Further research of this type is warranted to comprehensively understand the influence of the pandemic. Although both residents and program directors have a high consistency on clinical training, educational modifications and workforce restructuring issue, there are still some discrepancies. In a study discussing the impact on interventional cardiology, majority of residents believed that the pandemic affected their procedural competency but nearly all of the respond program directors believed that their residents would be ready for independent practice after completing the training year [56]. Another study collected narrative responses the program directors also revealed a more positive impressions on impact of COVID-19 on residency training [57]. Although there are some discrepancies on clinical competency, both residents and program directors believe that the pandemic would not affect their ability to pass the board examination and it is not necessary to extend the training course [39].

Because insufficient training was a concern, some assisted teaching methods were suggested and attempted by medical educators and clinical teachers. These assisted methods included simulations, online courses, the flipped classroom approach, and virtual reality/augmented reality [22, 75, 76]. Although these methods were adopted by many training program directors to compensate for the decreased clinical exposure and practice, the effect of doing so remained unknown because comparing learning outcomes between residency training in prepandemic and pandemic periods was impossible at this time. Extending training courses and delaying specialty board exams were considered options, but doing so could affect career planning and the quality of life of residents. The articles related to assisted teaching methods and adjustments were not included in our analysis because most of them were review articles rather than original works and failed to provide data regarding the effect. Although assisted teaching methods and adjusting training courses appear necessary, research must be conducted to determine the effectiveness of these methods.

### Limitations

This study has some limitations. First, we only selected articles published in English, and some important information published in different languages may have been overlooked. Second, articles included in our study were mainly published in American and European countries. Lack of data from other regions, even those severely affected by the pandemic, could render the conclusion insufficiently comprehensive. Third, the articles included in our study were within 1 year of the COVID-19 pandemic, and some long-term effects of this pandemic may not be evident in this time period. Finally, no article compared the learning outcomes of residents between prepandemic and pandemic periods. The final performance of these residents remains unknown. Further studies are necessary to determine the learning outcomes of residency training during this pandemic.

## Conclusions

The COVID-19 pandemic has greatly affected residency training globally, particularly surgical and interventional medical fields. Decreased clinical experience, reduced case volume, and disrupted education activities are major concerns in all fields. Although the publication of original studies investigating the effect of the COVID-19 pandemic on residency training is increasing, as of this writing, no study has compared the learning outcomes of residents between prepandemic and pandemic periods. Further study should be focused on the learning outcomes of residency training during the epidemic and evaluate the effectiveness of assisted teaching methods.

## Supplementary Information

**Additional file 1.** Information of the included original articles.

## Data Availability

The datasets generated and/or analysed during the current study are available from the corresponding author on reasonable request.

## Abbreviations

COVID-19: Coronavirus disease 2019
MEDLINE: Medical Literature Analysis and Retrieval System Online
EMBASE: Excerpta Medica database.
