# Supplementary material for: What is the impact of the COVID-19 pandemic on residency training: a systematic review and analysis
Source: BMC Med Educ. 2021 Dec 15;21:618. doi: 10.1186/s12909-021-03041-8 (PMC8671601; doi:10.1186/s12909-021-03041-8)
Supplement: Supplementary file 1 — Additional file 1. Information of the included original articles. [file 12909_2021_3041_MOESM1_ESM.docx]

**Additional file 1**. Information of the included original articles

| No | First author (Date) | Title | Journal |
| --- | --- | --- | --- |
| 1 | Abdul Hafiz Oladapo Adesunkanmi | Impact of the COVID-19 Pandemic on Surgical Residency Training: Perspective from a Low-Middle Income Country | World Journal of Surgery |
| 2 | Adel Salah Alahmadi | Residents' Perceived Impact of COVID-19 on Saudi Ophthalmology Training Programs-A Survey | Clinical Ophthalmology |
| 3 | Ahmad K Alhaj | Neurosurgery Residents' Perspective on COVID-19: Knowledge, Readiness, and Impact of this Pandemic | World Neurosurgery |
| 4 | Ahmed M Gabr | Diagnostic and Interventional Radiology Case Volume and Education in the Age of Pandemics: Impact Analysis and Potential Future Directions | Academic Radiology |
| 5 | Ameera Balhareth | Impact of COVID-19 pandemic on residency and fellowship training programs in Saudi Arabia: A nationwide cross-sectional study | Annals of Medicine and Surgery |
| 6 | Anne L Cravero | Impact of exposure to patients with COVID-19 on residents and fellows: an international survey of 1420 trainees | Postgraduate Medical Journal |
| 7 | Basil M Kahwash | Allergy/Immunology Trainee Experiences During the COVID-19 Pandemic: AAAAI Work Group Report of the Fellows-in-Training Committee | The Journal of Allergy and Clinical Immunology |
| 8 | Cesare Zoia | COVID-19 and neurosurgical training and education: an Italian perspective | Acta Neurochirurgica |
| 9 | Christoph Roemmele | Impact of the COVID-19 outbreak on endoscopy training in a tertiary care centre in Germany | Frontline Gastroenterology |
| 10 | Davide Pertile | The impact of COVID-19 pandemic on surgical residency programmes in Italy: a nationwide analysis on behalf of the Italian Polyspecialistic Young Surgeons Society (SPIGC) | Updates in Surgery |
| 11 | Deepak Mishra | The impact of COVID-19 related lockdown on ophthalmology training programs in India - Outcomes of a survey | Indian Journal of Ophthalmology |
| 12 | Devang Odedra | Impact of COVID-19 on Canadian Radiology Residency Training Programs | Canadian Association of Radiologists Journal |
| 13 | Dong-Gune Chang | The impact of COVID-19 pandemic on orthopaedic resident education: a nationwide survey study in South Korea | International Orthopaedics |
| 14 | E Christopher Ellison | Impact of the COVID-19 Pandemic on Surgical Training and Learner Well-Being: Report of a Survey of General Surgery and Other Surgical Specialty Educators | Journal of the American College of Surgeons |
| 15 | Edward J Caruana | Impact of coronavirus 2019 (COVID-19) on training and well-being in subspecialty surgery: A national survey of cardiothoracic trainees in the United Kingdom | The Journal of Thoracic and Cardiovascular Surgery |
| 16 | Erin M White | Surgical Education in the Time of COVID: Understanding the Early Response of Surgical Training Programs to the Novel Coronavirus Pandemic | Journal of Surgical Education |
| 17 | Francesco Bandi | Strategies to overcome limitations in Otolaryngology residency training during the COVID-19 pandemic | European Archives of Oto-Rhino-Laryngology |
| 18 | Garrett N Coyan | The impact of COVID-19 on thoracic surgery residency programs in the US: A program director survey | Journal of Cardiac Surgery |
| 19 | Gaurav Kumar Upadhyaya | Impact of COVID-19 on post-graduate orthopaedic training in Delhi-NCR | Journal of Clinical Orthopaedics and Trauma |
| 20 | Geoffrey H Rosen | Effect of COVID-19 on Urology Residency Training: A Nationwide Survey of Program Directors by the Society of Academic Urologists | The Journal of Urology |
| 21 | Giovanna Bitonti | Being an obstetrics and gynaecology resident during the COVID-19: Impact of the pandemic on the residency training program | The European Journal of Obstetrics & Gynecology and Reproductive Biology |
| 22 | Hassan Aziz | Effect of COVID-19 on Surgical Training Across the United States: A National Survey of General Surgery Residents | Journal of Surgical Education |
| 23 | Hsiang-Yun Lo | What is the impact of the COVID-19 pandemic on emergency medicine residency training: an observational study | BMC Medical Education |
| 24 | Jared Johnson | The impact of the COVID-19 pandemic on fellowship training: A national survey of pediatric otolaryngology fellowship directors | International Journal of Pediatric Otorhinolaryngology |
| 25 | Jessica B Robbins | COVID-19 Impact on Well-Being and Education in Radiology Residencies: A Survey of the Association of Program Directors in Radiology | Academic Radiology |
| 26 | Jian Zheng | General surgery chief residents' perspective on surgical education during the coronavirus disease 2019 (COVID-19) pandemic | Surgery |
| 27 | Johnathan A Khusid | Well-being and education of urology residents during the COVID-19 pandemic: Results of an American National Survey | International Journal of Clinical Practice |
| 28 | Joshua D Burks | Early Changes to Neurosurgery Resident Training During the COVID-19 Pandemic at a Large U.S. Academic Medical Center | World Neurosurgery |
| 29 | Julia R Coleman | COVID-19 Pandemic and the Lived Experience of Surgical Residents, Fellows, and Early-Career Surgeons in the American College of Surgeons | Journal of the American College of Surgeons |
| 30 | Katarzyna M Pawlak | Impact of COVID-19 on endoscopy trainees: an international survey | Gastrointestinal Endoscopy |
| 31 | Katherine E Fero | Perceived Impact of Urologic Surgery Training Program Modifications due to COVID-19 in the United States | Urology |
| 32 | Khurram Shahzad Khan | Impact of the COVID-19 pandemic on core surgical training | Scottish Medical Journal |
| 33 | Kofi Clarke | Impact of COVID-19 Pandemic on Training: Global Perceptions of Gastroenterology and Hepatology Fellows in the USA | Digestive Diseases and Sciences |
| 34 | Madhusudan Ganigara | Didactic education in paediatric cardiology during the COVID-19 pandemic: a national fellow survey | Cardiology in the Young |
| 35 | Mariantonia Ferrara | Reshaping ophthalmology training after COVID-19 pandemic | Eye |
| 36 | Medalit E Huamanchumo-Suyon | Impact of the COVID-19 pandemic on general surgery residency program in Peru: A cross-sectional study | Annals of Medicine and Surgery |
| 37 | Melissa K Meghpara | Repurposing a Small Community Hospital Surgical Residency Program in an Epicenter of the COVID-19 Pandemic | The American Journal of Surgery |
| 38 | Muhammad Osama | Impact of COVID-19 on surgical residency programs in Pakistan; A residents' perspective. Do programs need formal restructuring to adjust with the "new normal"? A cross-sectional survey study | International Journal of Surgery |
| 39 | Nahuel Paesano | Impact of COVID-19 Pandemic on Ibero-American Urology Residents: Perspective of American Confederation of Urology (CAU) | International Brazilian Journal of Urology |
| 40 | Natalie A Homer | Oculoplastic fellow education during the COVID-19 crisis | Orbit |
| 41 | Neo Poyiadji | COVID-19 and Radiology Resident Imaging Volumes–Differential Impact by Resident Training Year and Imaging Modality | Academic Radiology |
| 42 | Paloma Del C Monroig-Bosque | Pathology Trainee Redeployment and Education During the COVID-19 Pandemic: An Institutional Experience | Academic Pathology |
| 43 | Panayiotis D Megaloikonomos | Impact of the COVID-19 pandemic on orthopaedic and trauma surgery training in Europe | International Orthopaedics |
| 44 | Panayiotis E Pelargos | An Evaluation of Neurosurgical Resident Education and Sentiment During the Coronavirus Disease 2019 Pandemic: A North American Survey | World Neurosurgery |
| 45 | Raphael E Huntley | Early Effects of COVID-19 on Oral and Maxillofacial Surgery Residency Training-Results From a National Survey | Journal of Oral and Maxillofacial Surgery |
| 46 | Robert J Rothrock | By the Numbers Analysis of Effect of COVID-19 on a Neurosurgical Residency at the Epicenter | World Neurosurgery |
| 47 | S Veerasuri | Impact of COVID-19 on UK radiology training: a questionnaire study | Clinical Radiology |
| 48 | Samit Shah | Impact of the COVID-19 pandemic on interventional cardiology training in the United States | Catheterization and Cardiovascular Interventions |
| 49 | Tanush Gupta | Impact of the COVID-19 pandemic on interventional cardiology fellowship training in the New York metropolitan area: A perspective from the United States epicenter | Catheterization and Cardiovascular Interventions |
| 50 | Theresa Guo | Impact of the COVID-19 pandemic on Otolaryngology trainee education | Head & Neck |
| 51 | Tonya W An | How Are Orthopaedic Surgery Residencies Responding to the COVID-19 Pandemic? An Assessment of Resident Experiences in Cities of Major Virus Outbreak | Journal of the American Academy of Orthopaedic Surgeons |
| 52 | Virginia K Singla | The Impact of the COVID-19 Pandemic on Cardiac Electrophysiology Training: A Survey Study | Journal of Cardiovascular Electrophysiology |
| 53 | Zaid S Aljuboori | Early Effects of COVID-19 Pandemic on Neurosurgical Training in the United States: A Case Volume Analysis of 8 Programs | World Neurosurgery |
